# 1-(2,3-Di­hydroxy­prop­yl)-4-{2-[4-(di­methyl­amino)­phen­yl]vin­yl}pyridinium chloride

**DOI:** 10.1107/S1600536813033254

**Published:** 2013-12-21

**Authors:** Graeme J. Gainsford, M. Delower H. Bhuiyan, Andrew J. Kay

**Affiliations:** a Callaghan Innovation, PO Box 31-310, Lower Hutt, New Zealand

## Abstract

The title compound, C_18_H_23_N_2_O_2_^+^·Cl^−^, crystallizes with two independent cations and anions per cell. Each cation has twofold rotational disorder about the linking vinyl groups but with unequal occupancies [0.963 (5):0.037 (5) and 0.860 (8):0.140 (8)]. The two independent cations are close to being related by an inversion centre but the data does not support the expected centrosymmetric space-group assignment. The conclusion is that the differing rotational disorder has lead to an overall non-centrosymmetric lattice. In the crystal, the mol­ecules pack in layers parallel to (133) and (-13-3), chain-linked with motif *C* ^1^_2_(7) by the di­hydroxy­propyl O–H⋯Cl⋯H–O hydrogen bonds. Other lattice binding is provided by O—H⋯Cl, C—H⋯Cl and C—H⋯N inter­actions.

## Related literature   

For applications of organic push–pull chromophores, see: Kay *et al.* (2004 ▶); Bass *et al.* (2001 ▶); Prasad *et al.* (1988 ▶). For a related example of rotational disorder, see: Moreno-Fuquen *et al.* (2009 ▶). For details of the synthesis, see: Kay *et al.* (2001 ▶). For hydrogen-bond motifs, see: Bernstein *et al.* (1995 ▶).
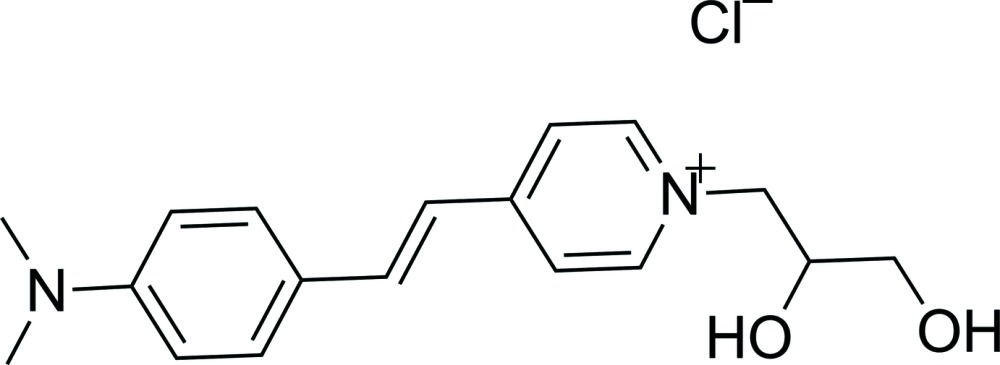


## Experimental   

### 

#### Crystal data  

C_18_H_23_N_2_O_2_^+^·Cl^−^
*M* *_r_* = 334.83
Monoclinic, 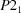

*a* = 8.4452 (3) Å
*b* = 13.2433 (5) Å
*c* = 15.3649 (6) Å
β = 100.077 (3)°
*V* = 1691.94 (11) Å^3^
*Z* = 4
Mo *K*α radiation
μ = 0.24 mm^−1^
*T* = 123 K
0.55 × 0.10 × 0.09 mm

#### Data collection  

Bruker–Nonius APEXII CCD area-detector diffractometer
Absorption correction: multi-scan (Blessing, 1995 ▶) *T*_min_ = 0.651, *T*_max_ = 0.746
38193 measured reflections
7581 independent reflections
5545 reflections with *I* > 2σ(*I*)
*R*_int_ = 0.063

#### Refinement  

*R*[*F* ^2^ > 2σ(*F* ^2^)] = 0.042
*wR*(*F* ^2^) = 0.096
*S* = 1.02
7581 reflections
474 parameters
13 restraints
H atoms treated by a mixture of independent and constrained refinement
Δρ_max_ = 0.18 e Å^−3^
Δρ_min_ = −0.20 e Å^−3^
Absolute structure: Flack parameter determined using 2152 quotients [(*I* ^+^)−(*I* ^−^)]/[(*I* ^+^)+(*I* ^−^)] (Parsons *et al.*, 2013 ▶)
Absolute structure parameter: 0.07 (4)

### 

Data collection: *APEX2* (Bruker, 2008 ▶); cell refinement: *SAINT* (Bruker, 2008 ▶); data reduction: *SAINT*; program(s) used to solve structure: *SHELXS97* (Sheldrick, 2008 ▶); program(s) used to refine structure: *SHELXL2012* (Sheldrick, 2008 ▶); molecular graphics: *ORTEP-3 for Windows* (Farrugia, 2012 ▶) and *Mercury* (Macrae *et al.*, 2006 ▶); software used to prepare material for publication: *SHELXL97*, *PLATON* (Spek, 2009 ▶) and *Mercury*.

## Supplementary Material

Crystal structure: contains datablock(s) global, I. DOI: 10.1107/S1600536813033254/sj5376sup1.cif

Structure factors: contains datablock(s) I. DOI: 10.1107/S1600536813033254/sj5376Isup2.hkl

Supporting information file. DOI: 10.1107/S1600536813033254/sj5376Isup3.cml

Additional supporting information:  crystallographic information; 3D view; checkCIF report

## Figures and Tables

**Table 1:** Hydrogen-bond geometry (Å, °)

*D*—H⋯*A*	*D*—H	H⋯*A*	*D*⋯*A*	*D*—H⋯*A*
O1—H1*O*⋯Cl1	0.83 (3)	2.20 (3)	3.024 (3)	174 (4)
O2—H2*O*⋯Cl1^i^	0.83 (3)	2.27 (3)	3.086 (3)	169 (4)
C2—H2⋯O2′^i^	0.95	2.40	3.222 (5)	145
C5*A*—H5*A*⋯Cl2^ii^	0.95	2.78	3.687 (5)	159
C8—H8*A*⋯N2^ii^	0.99	2.65	3.637 (5)	176
O1′—H1*O*′⋯Cl2	0.84 (3)	2.25 (3)	3.083 (3)	171 (4)
O2′—H2*O*′⋯Cl2^iii^	0.83 (3)	2.29 (3)	3.104 (3)	169 (4)
C6′—H6′⋯O2^iii^	0.95	2.40	3.236 (6)	147

Symmetry codes: (i) 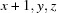; (ii) 
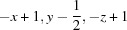
; (iii) 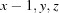.

**Fig. 1.**
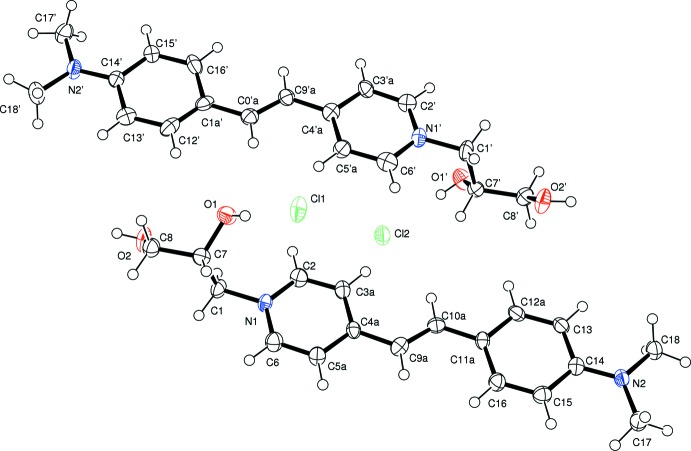
Labelling of the major(a) conformer cations and anions of the title compound, with 50% thermal ellipsoids (Farrugia, 2012).

**Fig. 2.**
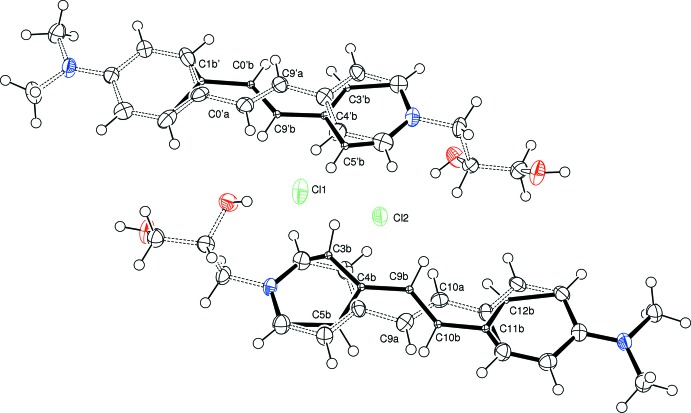
The total contents of the asymmetric unit, as for Fig. 1, with the minor cation rotamer (labelled b atoms) shown with filled bonds and major rotamer atoms (labelled a) connected with dotted bonds. The centre of the rotation is shown by inclusion of the C9 & C10 (a & b)labels.

**Fig. 3.**
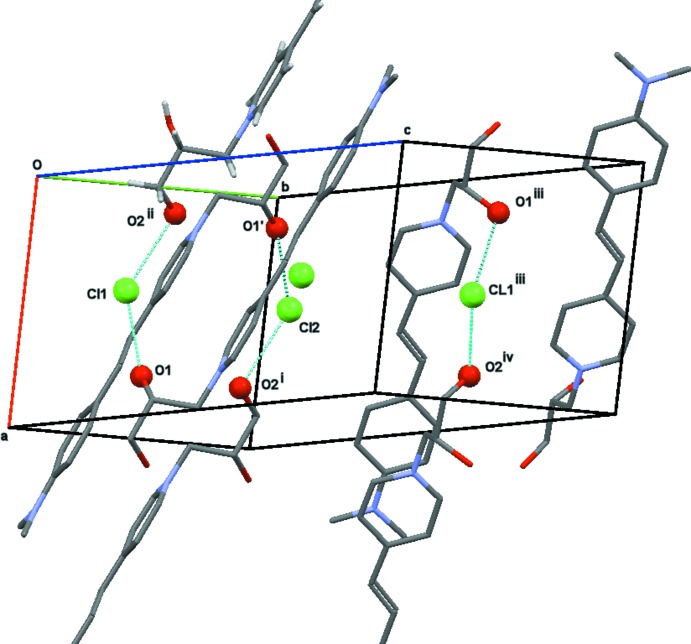
Packing diagram of (I) without H atoms for clarity. Hydrogen bonds shown as dashed blue lines (Mercury; Macrae et al., 2006). Symmetry codes: (i) 1 + x, y, z (ii) -1 + x, y, z (iii) 1 - x, 1/2 + y, 1 - z (iv) 2 - x, 1/2 + y, 1 - z.

## supplementary crystallographic information

### 1. Comment

Organic push–pull chromophores with large second-order nonlinear optical (NLO) properties are in demand due to their potential applications in photonic devices and optical information processing (Kay *et al.*, 2004; Bass *et al.*, 2001; Prasad *et al.*, 1988). A significant number of organic compounds available in the literature with large molecular NLO responses contain *N*,*N*-disubstituted anilines as this nucleus is an excellent electron donor. Due to the lack of tethering functionality of these chromophores, the possibility of synthesizing polymer containing chromophores is restricted. In this work, we have synthesized a new NLO chromophore containing an *N*,*N*-dimethyl aniline donor and an acceptor based on the dihydroxypropyl pyridinium chloride. The dihydroxypropyl substituent on the acceptor pyridinium nucleus will allow for covalent attachment of the molecule to a polymer backbone.

The asymmetric unit of the title compound (I) contains two independent 1-(2,3-dihydroxy-propyl)-4-[2-(4-dimethylamino-phenyl)-vinyl]-pyridinium cations (with primed and unprimed labels) and chloride anions almost related by an inversion centre (Fig. 1) in space group *P*2_1_. The screw axis and the c glide defining data number, average intensities and ratio of intensity/standard deviations were 61, 1/5, 0.4 and 906, 1.9, 3.1 respectively. The corresponding centosymmetric, and more usual, space group found for these compounds, *P*2_1_/*c* was rejected through the small but significant presence of the required glide plane absences; this was confirmed in attempted least squares refinements. At the conclusion of both space group refinements, the difference Fourier maps show the presence of two partially occupied rotational conformers about the alkene atoms (C9═C10, C9'═ C10') apparently confined to the vinyl linking atoms, also related by the same inversion centre (Fig. 2). Inclusion of the carbon atoms located on the Fourier difference map (see experimental) improved the agreement factors by about ~0.7% and results in a featureless difference map. Such rotational disorder is not uncommon amongst compounds containing C═C and C═N linkages: for example see Moreno-Fuquen *et al.* (2009).

The two cations (Fig 1) are very similar with RMS fits of 0.020 Å and 1.28° (*PLATON*, Spek, 2009); excluding the dihydroxy end groups, they are approximately planar (maximum deviations from the 18 atom planes are 0.055 (4) & 0.057 (5) Å for atoms C16 & C16') corresponding to the angle between the phenyl and pyridinium rings of 1.6 (2) & 2.8 (2)° for the unprimed and primed cations respectively.

The molecules pack in layers parallel to (133) and (133 planes), chain-linked by dihydroxypropyl O—H···Cl···H—O hydrogen bonds with motif *C*^1^_2_(7) (Bernstein *et al.*, 1995). Other lattice binding is provided by O—H···Cl and C—H···Cl, C—H···N interactions (Table 1).

### 2. Experimental

We synthesized the title compound by following the procedure in Kay *et al.* (2001). Single crystals were grown by slow ethyl acetate diffusion into a methanol solution of the compound.

### 3. Refinement

Indications from the final solution were that the structure could be refined in the centrosymmetric space group P21/c (*viz*. 95% in agreeement according to a *PLATON* analysis (Spek, 2009), with each molecule except atoms O1 & O1' related by inversion symmetry). The dataset does not support this with 984 glide plane systematic absences found to be weakly but significantly present out of the total set of 38305. Our experience with these planar NLO molecules is that they frequently form crystals with centrosymmetrically related molecules. In addition at the R1 value of 0.049, residual peaks in the same plane as the target molecules formed recognizable partial occupancy two fold rotational cation atoms, about the C9═C10 & C9'═C10' linkages (Figure 2).

The defined atoms were paired with the corresponding two major conformer cation atoms (a & b labels) and corresponding H atoms added in calculated positions where observed on the difference maps. H atoms on the located phenyl pyridinium C atoms were not resolved and so were fixed in calculated positions. All non-hydrogen atoms in the partially occupied minor rotamers were given a single group isotropic thermal parameter, which refined to 0.005 (3) A^2^. It was possible to model a chemically reasonable model using the *SHELXL* SAME controlling parameters (but only) with the pyridinium and phenyl rings treated independently & occupancies fixed. We elected to remain with the linked group occupancy refinement model presented here. Using SADI, the bond lengths C9═C10, C4–C9, C10–C11,C11—C12 and O–H were restrained to the same lengths. The final occupancies for major(A): minor(B) rotamers were 0.963 (5):0.037 (5)(unprimed a:b) and 0.860 (8):0.140 (8) (primed a:b).

Six reflections with *F_o_*<<Fc at low angle were omitted on the basis of background scatter and 2 were OMITted as outliers with Δ|(F_o_^2^-F_c_^2^)|/σ(F_o_^2^) > 4.5. A l l carbon-bound H atoms were constrained to their expected geometries [C—H 0.95,0.98, 0.99 Å] and refined with *U*_iso_ 1.2 times the *U*_eq_ of their parent atom except for the minor rotamer(*b*) H atoms with *U*_iso_=1.5*U*_eq_ of their parent atom. All other non-hydrogen atoms were refined with anisotropic thermal parameters.

### Crystal data

**Table:**

C_18_H_23_N_2_O_2_^+^·Cl^−^	*F*(000) = 712
*M**_r_* = 334.83	*D*_x_ = 1.314 Mg m^−^^3^
Monoclinic, *P*2_1_	Mo *K*α radiation, λ = 0.71073 Å
*a* = 8.4452 (3) Å	Cell parameters from 4999 reflections
*b* = 13.2433 (5) Å	θ = 2.7–23.4°
*c* = 15.3649 (6) Å	µ = 0.24 mm^−^^1^
β = 100.077 (3)°	*T* = 123 K
*V* = 1691.94 (11) Å^3^	Needle, red
*Z* = 4	0.55 × 0.10 × 0.09 mm

### Data collection

**Table:**

Bruker–Nonius APEXII CCD area-detector diffractometer	7581 independent reflections
Radiation source: fine-focus sealed tube	5545 reflections with *I* > 2σ(*I*)
Graphite monochromator	*R*_int_ = 0.063
Detector resolution: 8.333 pixels mm^-1^	θ_max_ = 27.3°, θ_min_ = 2.6°
φ and ω scans	*h* = −10→10
Absorption correction: multi-scan (Blessing, 1995)	*k* = −17→17
*T*_min_ = 0.651, *T*_max_ = 0.746	*l* = −19→19
38193 measured reflections	

### Refinement

**Table:**

Refinement on *F*^2^	Hydrogen site location: inferred from neighbouring sites
Least-squares matrix: full	H atoms treated by a mixture of independent and constrained refinement
*R*[*F*^2^ > 2σ(*F*^2^)] = 0.042	*w* = 1/[σ^2^(*F*_o_^2^) + (0.0362*P*)^2^ + 0.2405*P*] where *P* = (*F*_o_^2^ + 2*F*_c_^2^)/3
*wR*(*F*^2^) = 0.096	(Δ/σ)_max_ = 0.001
*S* = 1.02	Δρ_max_ = 0.18 e Å^−^^3^
7581 reflections	Δρ_min_ = −0.20 e Å^−^^3^
474 parameters	Extinction correction: *SHELXL*, Fc^*^=kFc[1+0.001xFc^2^λ^3^/sin(2θ)]^-1/4^
13 restraints	Extinction coefficient: 0.0039 (10)
Primary atom site location: structure-invariant direct methods	Absolute structure: Flack parameter determined using 2152 quotients [(*I*^+^)-(*I*^-^)]/[(*I*^+^)+(*I*^-^)] (Parsons *et al.*, 2013)
Secondary atom site location: difference Fourier map	Absolute structure parameter: 0.07 (4)

### Special details

**Table:**

Geometry. All e.s.d.'s (except the e.s.d. in the dihedral angle between two l.s. planes) are estimated using the full covariance matrix. The cell e.s.d.'s are taken into account individually in the estimation of e.s.d.'s in distances, angles and torsion angles; correlations between e.s.d.'s in cell parameters are only used when they are defined by crystal symmetry. An approximate (isotropic) treatment of cell e.s.d.'s is used for estimating e.s.d.'s involving l.s. planes.

### Fractional atomic coordinates and isotropic or equivalent isotropic displacement parameters (Å^2^)

**Table:**

	*x*	*y*	*z*	*U*_iso_*/*U*_eq_	Occ. (<1)
Cl1	0.46974 (12)	0.13741 (8)	0.18879 (7)	0.0391 (3)	
O1	0.8050 (3)	0.2226 (2)	0.20024 (17)	0.0340 (6)	
H1O	0.716 (4)	0.196 (3)	0.200 (3)	0.041*	
O2	1.1625 (3)	0.2328 (2)	0.2362 (2)	0.0367 (8)	
H2O	1.241 (4)	0.211 (4)	0.216 (3)	0.044*	
N1	0.8014 (4)	0.2692 (2)	0.3860 (2)	0.0238 (8)	
N2	−0.2944 (4)	0.4615 (3)	0.6126 (2)	0.0267 (8)	
C1	0.9463 (5)	0.2493 (3)	0.3480 (3)	0.0283 (10)	
H1A	1.0312	0.2210	0.3942	0.034*	
H1B	0.9866	0.3135	0.3272	0.034*	
C2	0.6990 (6)	0.3426 (3)	0.3522 (3)	0.0291 (10)	
H2	0.7222	0.3826	0.3046	0.035*	
C3A	0.5613 (6)	0.3606 (4)	0.3857 (3)	0.0273 (11)	0.963 (5)
H3A	0.4895	0.4122	0.3606	0.033*	0.963 (5)
C4A	0.5256 (6)	0.3031 (4)	0.4569 (3)	0.0224 (10)	0.963 (5)
C5A	0.6362 (6)	0.2279 (4)	0.4900 (3)	0.0272 (12)	0.963 (5)
H5A	0.6177	0.1873	0.5382	0.033*	0.963 (5)
C6	0.7688 (5)	0.2128 (3)	0.4541 (3)	0.0306 (10)	
H6	0.8417	0.1608	0.4773	0.037*	
C7	0.9110 (4)	0.1751 (3)	0.2706 (2)	0.0257 (9)	
H7	0.8584	0.1133	0.2898	0.031*	
C8	1.0658 (5)	0.1459 (3)	0.2410 (3)	0.0298 (10)	
H8A	1.1251	0.0969	0.2833	0.036*	
H8B	1.0416	0.1132	0.1822	0.036*	
C9A	0.3844 (5)	0.3174 (3)	0.4975 (3)	0.0274 (11)	0.963 (5)
H9A	0.3703	0.2733	0.5444	0.033*	0.963 (5)
C10A	0.2722 (5)	0.3887 (3)	0.4733 (3)	0.0243 (11)	0.963 (5)
H10A	0.2872	0.4319	0.4261	0.029*	0.963 (5)
C11A	0.1307 (5)	0.4060 (4)	0.5123 (3)	0.0221 (10)	0.963 (5)
C12A	0.0194 (6)	0.4797 (4)	0.4758 (3)	0.0262 (11)	0.963 (5)
H12A	0.0398	0.5175	0.4264	0.031*	0.963 (5)
C13	−0.1171 (5)	0.4989 (3)	0.5090 (3)	0.0257 (10)	
H13	−0.1878	0.5506	0.4829	0.031*	
C14	−0.1560 (5)	0.4442 (3)	0.5807 (3)	0.0239 (9)	
C15	−0.0453 (5)	0.3693 (3)	0.6177 (3)	0.0287 (10)	
H15	−0.0666	0.3308	0.6665	0.034*	
C16	0.0916 (5)	0.3513 (3)	0.5845 (3)	0.0288 (10)	
H16	0.1632	0.3002	0.6109	0.035*	
C17	−0.3366 (5)	0.4042 (3)	0.6852 (3)	0.0317 (10)	
H17A	−0.3928	0.3423	0.6624	0.038*	
H17B	−0.4070	0.4447	0.7157	0.038*	
H17C	−0.2387	0.3865	0.7266	0.038*	
C18	−0.3998 (5)	0.5442 (3)	0.5770 (3)	0.0305 (10)	
H18A	−0.3436	0.6085	0.5903	0.037*	
H18B	−0.4968	0.5432	0.6038	0.037*	
H18C	−0.4300	0.5363	0.5128	0.037*	
Cl2	0.52229 (11)	0.62109 (8)	0.32090 (6)	0.0353 (3)	
O1'	0.1812 (3)	0.67503 (19)	0.22476 (18)	0.0358 (6)	
H1O'	0.276 (3)	0.668 (3)	0.251 (2)	0.043*	
O2'	−0.1666 (4)	0.5388 (2)	0.2646 (2)	0.0375 (8)	
H2O'	−0.256 (4)	0.558 (3)	0.273 (3)	0.045*	
N1'	0.1959 (4)	0.5059 (2)	0.1129 (2)	0.0265 (8)	
N2'	1.2916 (4)	0.3085 (3)	−0.1111 (2)	0.0301 (9)	
C1'	0.0508 (5)	0.5298 (4)	0.1502 (3)	0.0315 (10)	
H1C	−0.0047	0.4663	0.1606	0.038*	
H1D	−0.0237	0.5705	0.1070	0.038*	
C2'	0.2335 (5)	0.5609 (3)	0.0466 (3)	0.0299 (10)	
H2'	0.1642	0.6153	0.0250	0.036*	
C3'A	0.3618 (6)	0.5448 (5)	0.0084 (4)	0.0272 (14)	0.860 (8)
H3'A	0.3782	0.5843	−0.0409	0.033*	0.860 (8)
C4'A	0.4716 (6)	0.4694 (5)	0.0416 (4)	0.0255 (13)	0.860 (8)
C5'A	0.4347 (7)	0.4125 (5)	0.1115 (4)	0.0289 (13)	0.860 (8)
H5'A	0.5061	0.3603	0.1359	0.035*	0.860 (8)
C6'	0.2939 (5)	0.4303 (3)	0.1473 (3)	0.0344 (11)	
H6'	0.2691	0.3900	0.1943	0.041*	
C7'	0.0910 (5)	0.5876 (3)	0.2360 (3)	0.0240 (9)	
H7'	0.1530	0.5435	0.2828	0.029*	
C8'	−0.0630 (5)	0.6221 (3)	0.2639 (2)	0.0292 (9)	
H8C	−0.0390	0.6525	0.3236	0.035*	
H8D	−0.1158	0.6740	0.2223	0.035*	
C9'A	0.6120 (6)	0.4533 (4)	0.0014 (3)	0.0262 (13)	0.860 (8)
H9'A	0.6234	0.4949	−0.0475	0.031*	0.860 (8)
C0'A	0.7267 (6)	0.3854 (4)	0.0271 (3)	0.0277 (14)	0.860 (8)
H0'A	0.7135	0.3442	0.0759	0.033*	0.860 (8)
C1A'	0.8693 (7)	0.3668 (5)	−0.0108 (4)	0.0274 (16)	0.860 (8)
C12'	0.9755 (5)	0.2958 (4)	0.0236 (3)	0.0329 (11)	
H12'	0.9535	0.2580	0.0727	0.040*	
C13'	1.1137 (5)	0.2746 (3)	−0.0080 (3)	0.0300 (10)	
H13'	1.1835	0.2230	0.0192	0.036*	
C14'	1.1537 (5)	0.3282 (3)	−0.0805 (3)	0.0237 (9)	
C15'	1.0459 (5)	0.4038 (3)	−0.1190 (3)	0.0294 (10)	
H15'	1.0693	0.4419	−0.1675	0.035*	
C16'	0.9064 (5)	0.4225 (4)	−0.0862 (3)	0.0333 (11)	
H16'	0.8336	0.4726	−0.1134	0.040*	
C17'	1.3372 (5)	0.3656 (4)	−0.1837 (3)	0.0383 (12)	
H17D	1.2600	0.3524	−0.2380	0.046*	
H17E	1.4450	0.3451	−0.1921	0.046*	
H17F	1.3372	0.4379	−0.1700	0.046*	
C18'	1.3947 (5)	0.2250 (4)	−0.0766 (3)	0.0368 (11)	
H18D	1.4419	0.2383	−0.0148	0.044*	
H18E	1.4807	0.2174	−0.1115	0.044*	
H18F	1.3311	0.1627	−0.0803	0.044*	
C3'B	0.410 (4)	0.524 (2)	0.0343 (17)	0.006 (3)*	0.140 (8)
H3'B	0.4688	0.5617	−0.0024	0.008*	0.140 (8)
C4'B	0.479 (3)	0.439 (3)	0.077 (2)	0.006 (3)*	0.140 (8)
C5'B	0.413 (3)	0.3892 (19)	0.1394 (17)	0.006 (3)*	0.140 (8)
H5'B	0.4545	0.3292	0.1725	0.008*	0.140 (8)
C9'B	0.628 (2)	0.3994 (16)	0.0562 (14)	0.006 (3)*	0.140 (8)
H9'B	0.6753	0.3443	0.0912	0.008*	0.140 (8)
C0'B	0.709 (3)	0.4332 (18)	−0.0095 (16)	0.006 (3)*	0.140 (8)
H0'B	0.6634	0.4898	−0.0426	0.008*	0.140 (8)
C1B'	0.863 (4)	0.391 (2)	−0.035 (2)	0.006 (3)*	0.140 (8)
C3B	0.597 (12)	0.347 (7)	0.348 (6)	0.006 (3)*	0.037 (5)
H3B	0.5409	0.3627	0.2911	0.008*	0.037 (5)
C4B	0.505 (9)	0.332 (8)	0.418 (8)	0.006 (3)*	0.037 (5)
C5B	0.600 (15)	0.265 (9)	0.478 (7)	0.006 (3)*	0.037 (5)
H5B	0.5642	0.2460	0.5297	0.008*	0.037 (5)
C9B	0.351 (8)	0.361 (6)	0.440 (4)	0.006 (3)*	0.037 (5)
H9B	0.2787	0.3887	0.3921	0.008*	0.037 (5)
C10B	0.290 (9)	0.355 (6)	0.515 (5)	0.006 (3)*	0.037 (5)
H10B	0.3700	0.3428	0.5658	0.008*	0.037 (5)
C11B	0.130 (8)	0.364 (7)	0.537 (7)	0.006 (3)*	0.037 (5)
C12B	0.072 (12)	0.456 (7)	0.501 (6)	0.006 (3)*	0.037 (5)
H12B	0.1379	0.4973	0.4707	0.008*	0.037 (5)

### Atomic displacement parameters (Å^2^)

**Table:**

	*U*^11^	*U*^22^	*U*^33^	*U*^12^	*U*^13^	*U*^23^
Cl1	0.0256 (6)	0.0416 (7)	0.0534 (7)	−0.0007 (5)	0.0158 (5)	−0.0082 (6)
O1	0.0258 (14)	0.0429 (15)	0.0330 (15)	−0.0025 (12)	0.0042 (11)	0.0092 (12)
O2	0.0278 (17)	0.0325 (17)	0.055 (2)	−0.0010 (15)	0.0217 (15)	−0.0060 (16)
N1	0.0173 (18)	0.0271 (19)	0.029 (2)	−0.0026 (15)	0.0086 (14)	−0.0003 (16)
N2	0.0214 (19)	0.0282 (19)	0.031 (2)	0.0049 (16)	0.0057 (15)	0.0031 (17)
C1	0.021 (2)	0.036 (2)	0.030 (2)	−0.0027 (18)	0.0100 (18)	−0.0025 (19)
C2	0.031 (3)	0.022 (2)	0.034 (3)	0.0003 (19)	0.008 (2)	0.0018 (19)
C3A	0.024 (3)	0.027 (3)	0.031 (3)	0.003 (2)	0.005 (2)	−0.003 (2)
C4A	0.017 (2)	0.024 (3)	0.026 (3)	−0.001 (2)	0.0025 (19)	−0.003 (2)
C5A	0.026 (3)	0.024 (3)	0.033 (3)	0.000 (2)	0.008 (2)	0.002 (2)
C6	0.028 (2)	0.029 (2)	0.036 (3)	−0.0018 (19)	0.0085 (19)	0.001 (2)
C7	0.022 (2)	0.027 (2)	0.027 (2)	−0.0044 (18)	0.0031 (16)	0.0017 (18)
C8	0.029 (2)	0.029 (2)	0.033 (2)	0.0012 (19)	0.0091 (17)	0.002 (2)
C9A	0.028 (3)	0.026 (3)	0.029 (3)	−0.006 (2)	0.009 (2)	−0.004 (2)
C10A	0.027 (3)	0.023 (2)	0.022 (2)	−0.0025 (19)	0.0033 (19)	−0.001 (2)
C11A	0.018 (2)	0.021 (3)	0.028 (3)	−0.0019 (19)	0.0045 (19)	−0.003 (2)
C12A	0.023 (2)	0.029 (3)	0.024 (3)	0.0009 (19)	−0.0015 (19)	0.004 (2)
C13	0.023 (2)	0.027 (2)	0.025 (2)	0.0041 (18)	−0.0002 (18)	0.0015 (19)
C14	0.022 (2)	0.022 (2)	0.027 (2)	−0.0022 (17)	0.0044 (18)	−0.0050 (18)
C15	0.030 (2)	0.028 (2)	0.028 (2)	−0.0022 (19)	0.0061 (19)	0.002 (2)
C16	0.028 (2)	0.031 (2)	0.028 (2)	0.0039 (19)	0.006 (2)	−0.003 (2)
C17	0.029 (2)	0.032 (2)	0.037 (3)	0.004 (2)	0.011 (2)	0.005 (2)
C18	0.026 (2)	0.032 (2)	0.034 (3)	0.006 (2)	0.0063 (19)	−0.001 (2)
Cl2	0.0285 (5)	0.0390 (6)	0.0390 (6)	0.0064 (5)	0.0072 (4)	−0.0030 (5)
O1'	0.0276 (14)	0.0284 (14)	0.0486 (17)	−0.0042 (11)	−0.0008 (12)	0.0010 (12)
O2'	0.0325 (18)	0.0292 (17)	0.057 (2)	0.0004 (15)	0.0247 (16)	0.0018 (16)
N1'	0.0205 (18)	0.0269 (19)	0.033 (2)	−0.0015 (15)	0.0066 (15)	−0.0085 (17)
N2'	0.028 (2)	0.029 (2)	0.037 (2)	0.0055 (17)	0.0158 (17)	0.0049 (17)
C1'	0.018 (2)	0.042 (3)	0.036 (3)	−0.0042 (19)	0.0101 (18)	−0.009 (2)
C2'	0.032 (2)	0.027 (2)	0.031 (2)	−0.0012 (19)	0.0052 (19)	0.000 (2)
C3'A	0.019 (3)	0.031 (3)	0.030 (3)	−0.001 (2)	0.001 (2)	0.000 (2)
C4'A	0.022 (3)	0.025 (3)	0.030 (3)	−0.010 (2)	0.004 (2)	−0.006 (2)
C5'A	0.034 (3)	0.024 (3)	0.030 (4)	0.003 (2)	0.010 (3)	0.003 (2)
C6'	0.040 (3)	0.027 (2)	0.035 (3)	−0.001 (2)	0.002 (2)	−0.002 (2)
C7'	0.028 (2)	0.020 (2)	0.025 (2)	0.0014 (17)	0.0051 (17)	0.0024 (16)
C8'	0.033 (2)	0.025 (2)	0.030 (2)	0.001 (2)	0.0088 (17)	−0.002 (2)
C9'A	0.022 (3)	0.030 (3)	0.026 (3)	−0.002 (2)	0.002 (2)	−0.001 (2)
C0'A	0.026 (3)	0.032 (3)	0.025 (3)	−0.004 (2)	0.002 (2)	−0.003 (3)
C1A'	0.028 (3)	0.036 (4)	0.018 (3)	−0.007 (3)	0.004 (3)	−0.005 (3)
C12'	0.031 (3)	0.043 (3)	0.027 (2)	−0.012 (2)	0.012 (2)	−0.010 (2)
C13'	0.032 (3)	0.029 (2)	0.029 (2)	−0.007 (2)	0.005 (2)	−0.002 (2)
C14'	0.020 (2)	0.026 (2)	0.026 (2)	−0.0038 (17)	0.0037 (18)	−0.0053 (18)
C15'	0.027 (2)	0.033 (2)	0.029 (2)	0.0047 (19)	0.0053 (19)	0.000 (2)
C16'	0.022 (2)	0.034 (3)	0.041 (3)	0.0056 (19)	−0.004 (2)	−0.006 (2)
C17'	0.034 (3)	0.048 (3)	0.037 (3)	0.000 (2)	0.017 (2)	0.000 (2)
C18'	0.028 (2)	0.034 (3)	0.049 (3)	0.008 (2)	0.007 (2)	0.001 (2)

### Geometric parameters (Å, º)

**Table:**

O1—C7	1.424 (4)	C1'—C7'	1.511 (5)
O1—H1O	0.83 (3)	C1'—H1C	0.9900
O2—C8	1.420 (5)	C1'—H1D	0.9900
O2—H2O	0.83 (3)	C2'—C3'A	1.336 (6)
N1—C2	1.344 (5)	C2'—H2'	0.9500
N1—C6	1.352 (5)	C3'A—C4'A	1.397 (8)
N1—C1	1.469 (5)	C3'A—H3'A	0.9500
N2—C14	1.365 (5)	C4'A—C5'A	1.392 (7)
N2—C17	1.444 (5)	C4'A—C9'A	1.444 (7)
N2—C18	1.456 (5)	C5'A—C6'	1.414 (7)
C1—C7	1.531 (6)	C5'A—H5'A	0.9500
C1—H1A	0.9900	C6'—H6'	0.9500
C1—H1B	0.9900	C7'—C8'	1.510 (5)
C2—C3A	1.372 (6)	C7'—H7'	1.0000
C2—H2	0.9500	C8'—H8C	0.9900
C3A—C4A	1.409 (6)	C8'—H8D	0.9900
C3A—H3A	0.9500	C9'A—C0'A	1.331 (7)
C4A—C5A	1.398 (6)	C9'A—H9'A	0.9500
C4A—C9A	1.452 (6)	C0'A—C1A'	1.447 (8)
C5A—C6	1.348 (6)	C0'A—H0'A	0.9500
C5A—H5A	0.9500	C1A'—C12'	1.343 (8)
C6—H6	0.9500	C1A'—C16'	1.454 (7)
C7—C8	1.507 (5)	C12'—C13'	1.369 (6)
C7—H7	1.0000	C12'—H12'	0.9500
C8—H8A	0.9900	C13'—C14'	1.410 (6)
C8—H8B	0.9900	C13'—H13'	0.9500
C9A—C10A	1.344 (6)	C14'—C15'	1.411 (6)
C9A—H9A	0.9500	C15'—C16'	1.382 (6)
C10A—C11A	1.445 (6)	C15'—H15'	0.9500
C10A—H10A	0.9500	C16'—H16'	0.9500
C11A—C12A	1.402 (6)	C17'—H17D	0.9800
C11A—C16	1.412 (6)	C17'—H17E	0.9800
C12A—C13	1.363 (6)	C17'—H17F	0.9800
C12A—H12A	0.9500	C18'—H18D	0.9800
C13—C14	1.405 (5)	C18'—H18E	0.9800
C13—H13	0.9500	C18'—H18F	0.9800
C14—C15	1.412 (5)	C3'B—C4'B	1.38 (4)
C15—C16	1.364 (6)	C3'B—H3'B	0.95 (3)
C15—H15	0.9500	C4'B—C5'B	1.36 (4)
C16—H16	0.9500	C4'B—C9'B	1.45 (2)
C17—H17A	0.9800	C5'B—H5'B	0.98 (2)
C17—H17B	0.9800	C9'B—C0'B	1.39 (3)
C17—H17C	0.9800	C9'B—H9'B	0.9500
C18—H18A	0.9800	C0'B—C1B'	1.53 (4)
C18—H18B	0.9800	C0'B—H0'B	0.9500
C18—H18C	0.9800	C3B—C4B	1.44 (12)
O1'—C7'	1.413 (5)	C3B—H3B	0.94 (9)
O1'—H1O'	0.84 (3)	C4B—C5B	1.42 (14)
O2'—C8'	1.409 (5)	C4B—C9B	1.45 (3)
O2'—H2O'	0.83 (3)	C5B—H5B	0.93 (10)
N1'—C2'	1.336 (5)	C9B—C10B	1.35 (3)
N1'—C6'	1.347 (5)	C9B—H9B	0.9500
N1'—C1'	1.475 (5)	C10B—C11B	1.45 (3)
N2'—C14'	1.356 (5)	C10B—H10B	0.9500
N2'—C18'	1.449 (5)	C11B—C12B	1.39 (3)
N2'—C17'	1.455 (5)	C12B—H12B	0.95 (9)
			
C7—O1—H1O	105 (3)	C7'—C1'—H1D	109.2
C8—O2—H2O	104 (3)	H1C—C1'—H1D	107.9
C2—N1—C6	119.6 (4)	N1'—C2'—C3'A	124.6 (5)
C2—N1—C1	120.0 (3)	N1'—C2'—H2'	117.7
C6—N1—C1	120.3 (4)	C3'A—C2'—H2'	117.7
C14—N2—C17	122.1 (3)	C2'—C3'A—C4'A	119.5 (5)
C14—N2—C18	119.8 (3)	C2'—C3'A—H3'A	120.3
C17—N2—C18	118.0 (3)	C4'A—C3'A—H3'A	120.3
N1—C1—C7	111.2 (3)	C5'A—C4'A—C3'A	116.4 (5)
N1—C1—H1A	109.4	C5'A—C4'A—C9'A	124.2 (6)
C7—C1—H1A	109.4	C3'A—C4'A—C9'A	119.4 (5)
N1—C1—H1B	109.4	C4'A—C5'A—C6'	121.8 (5)
C7—C1—H1B	109.4	C4'A—C5'A—H5'A	119.1
H1A—C1—H1B	108.0	C6'—C5'A—H5'A	119.1
N1—C2—C3A	120.7 (4)	N1'—C6'—C5'A	118.2 (5)
N1—C2—H2	119.6	N1'—C6'—H6'	120.9
C3A—C2—H2	119.6	C5'A—C6'—H6'	120.9
C2—C3A—C4A	120.6 (4)	O1'—C7'—C8'	107.3 (3)
C2—C3A—H3A	119.7	O1'—C7'—C1'	110.6 (3)
C4A—C3A—H3A	119.7	C8'—C7'—C1'	109.1 (3)
C5A—C4A—C3A	116.6 (4)	O1'—C7'—H7'	109.9
C5A—C4A—C9A	118.9 (5)	C8'—C7'—H7'	109.9
C3A—C4A—C9A	124.5 (5)	C1'—C7'—H7'	109.9
C6—C5A—C4A	120.4 (4)	O2'—C8'—C7'	109.4 (3)
C6—C5A—H5A	119.8	O2'—C8'—H8C	109.8
C4A—C5A—H5A	119.8	C7'—C8'—H8C	109.8
C5A—C6—N1	122.1 (4)	O2'—C8'—H8D	109.8
C5A—C6—H6	119.0	C7'—C8'—H8D	109.8
N1—C6—H6	119.0	H8C—C8'—H8D	108.2
O1—C7—C8	110.3 (3)	C0'A—C9'A—C4'A	125.6 (5)
O1—C7—C1	108.5 (3)	C0'A—C9'A—H9'A	117.2
C8—C7—C1	109.7 (3)	C4'A—C9'A—H9'A	117.2
O1—C7—H7	109.4	C9'A—C0'A—C1A'	127.6 (6)
C8—C7—H7	109.4	C9'A—C0'A—H0'A	116.2
C1—C7—H7	109.4	C1A'—C0'A—H0'A	116.2
O2—C8—C7	110.2 (3)	C12'—C1A'—C0'A	120.5 (5)
O2—C8—H8A	109.6	C12'—C1A'—C16'	116.5 (5)
C7—C8—H8A	109.6	C0'A—C1A'—C16'	123.0 (6)
O2—C8—H8B	109.6	C1A'—C12'—C13'	123.8 (5)
C7—C8—H8B	109.6	C1A'—C12'—H12'	118.1
H8A—C8—H8B	108.1	C13'—C12'—H12'	118.1
C10A—C9A—C4A	124.4 (5)	C12'—C13'—C14'	120.9 (4)
C10A—C9A—H9A	117.8	C12'—C13'—H13'	119.6
C4A—C9A—H9A	117.8	C14'—C13'—H13'	119.6
C9A—C10A—C11A	126.2 (5)	N2'—C14'—C15'	121.4 (4)
C9A—C10A—H10A	116.9	N2'—C14'—C13'	121.1 (4)
C11A—C10A—H10A	116.9	C15'—C14'—C13'	117.5 (4)
C12A—C11A—C16	116.0 (4)	C16'—C15'—C14'	120.2 (4)
C12A—C11A—C10A	119.3 (5)	C16'—C15'—H15'	119.9
C16—C11A—C10A	124.6 (5)	C14'—C15'—H15'	119.9
C13—C12A—C11A	122.1 (4)	C15'—C16'—C1A'	121.0 (4)
C13—C12A—H12A	118.9	C15'—C16'—H16'	119.5
C11A—C12A—H12A	118.9	C1A'—C16'—H16'	119.5
C12A—C13—C14	121.7 (4)	N2'—C17'—H17D	109.5
C12A—C13—H13	119.1	N2'—C17'—H17E	109.5
C14—C13—H13	119.1	H17D—C17'—H17E	109.5
N2—C14—C13	121.9 (4)	N2'—C17'—H17F	109.5
N2—C14—C15	121.4 (4)	H17D—C17'—H17F	109.5
C13—C14—C15	116.7 (4)	H17E—C17'—H17F	109.5
C16—C15—C14	121.1 (4)	N2'—C18'—H18D	109.5
C16—C15—H15	119.5	N2'—C18'—H18E	109.5
C14—C15—H15	119.5	H18D—C18'—H18E	109.5
C15—C16—C11A	122.3 (4)	N2'—C18'—H18F	109.5
C15—C16—H16	118.8	H18D—C18'—H18F	109.5
C11A—C16—H16	118.8	H18E—C18'—H18F	109.5
N2—C17—H17A	109.5	C4'B—C3'B—H3'B	119 (3)
N2—C17—H17B	109.5	C5'B—C4'B—C3'B	123 (2)
H17A—C17—H17B	109.5	C5'B—C4'B—C9'B	117 (3)
N2—C17—H17C	109.5	C3'B—C4'B—C9'B	120 (3)
H17A—C17—H17C	109.5	C4'B—C5'B—H5'B	127 (3)
H17B—C17—H17C	109.5	C0'B—C9'B—C4'B	127 (2)
N2—C18—H18A	109.5	C0'B—C9'B—H9'B	116.5
N2—C18—H18B	109.5	C4'B—C9'B—H9'B	116.5
H18A—C18—H18B	109.5	C9'B—C0'B—C1B'	128 (2)
N2—C18—H18C	109.5	C9'B—C0'B—H0'B	115.8
H18A—C18—H18C	109.5	C1B'—C0'B—H0'B	115.8
H18B—C18—H18C	109.5	C4B—C3B—H3B	118 (10)
C7'—O1'—H1O'	110 (3)	C5B—C4B—C3B	105 (7)
C8'—O2'—H2O'	111 (3)	C5B—C4B—C9B	116 (10)
C2'—N1'—C6'	119.5 (4)	C3B—C4B—C9B	139 (10)
C2'—N1'—C1'	120.3 (4)	C4B—C5B—H5B	119 (10)
C6'—N1'—C1'	120.2 (4)	C10B—C9B—C4B	133 (8)
C14'—N2'—C18'	121.2 (4)	C10B—C9B—H9B	113.4
C14'—N2'—C17'	121.6 (4)	C4B—C9B—H9B	113.4
C18'—N2'—C17'	117.1 (3)	C9B—C10B—C11B	135 (8)
N1'—C1'—C7'	111.9 (3)	C9B—C10B—H10B	112.6
N1'—C1'—H1C	109.2	C11B—C10B—H10B	112.6
C7'—C1'—H1C	109.2	C12B—C11B—C10B	105 (8)
N1'—C1'—H1D	109.2	C11B—C12B—H12B	120 (10)
			
C2—N1—C1—C7	−87.1 (4)	N1'—C2'—C3'A—C4'A	−3.7 (8)
C6—N1—C1—C7	91.8 (4)	C2'—C3'A—C4'A—C5'A	2.5 (7)
C6—N1—C2—C3A	−0.4 (6)	C2'—C3'A—C4'A—C9'A	−178.7 (4)
C1—N1—C2—C3A	178.4 (4)	C3'A—C4'A—C5'A—C6'	−0.2 (7)
N1—C2—C3A—C4A	0.8 (7)	C9'A—C4'A—C5'A—C6'	−178.8 (5)
C2—C3A—C4A—C5A	−0.4 (6)	C2'—N1'—C6'—C5'A	0.4 (6)
C2—C3A—C4A—C9A	179.1 (4)	C1'—N1'—C6'—C5'A	−177.7 (4)
C3A—C4A—C5A—C6	−0.3 (7)	C4'A—C5'A—C6'—N1'	−1.3 (7)
C9A—C4A—C5A—C6	−179.8 (4)	N1'—C1'—C7'—O1'	54.5 (5)
C4A—C5A—C6—N1	0.6 (7)	N1'—C1'—C7'—C8'	172.2 (3)
C2—N1—C6—C5A	−0.3 (6)	O1'—C7'—C8'—O2'	172.2 (3)
C1—N1—C6—C5A	−179.2 (4)	C1'—C7'—C8'—O2'	52.4 (4)
N1—C1—C7—O1	66.8 (4)	C5'A—C4'A—C9'A—C0'A	−2.3 (8)
N1—C1—C7—C8	−172.6 (3)	C3'A—C4'A—C9'A—C0'A	179.0 (5)
O1—C7—C8—O2	75.1 (4)	C4'A—C9'A—C0'A—C1A'	−179.8 (5)
C1—C7—C8—O2	−44.4 (5)	C9'A—C0'A—C1A'—C12'	179.2 (5)
C5A—C4A—C9A—C10A	178.0 (4)	C9'A—C0'A—C1A'—C16'	−0.6 (9)
C3A—C4A—C9A—C10A	−1.5 (7)	C0'A—C1A'—C12'—C13'	−179.4 (5)
C4A—C9A—C10A—C11A	−179.5 (4)	C16'—C1A'—C12'—C13'	0.4 (8)
C9A—C10A—C11A—C12A	−176.0 (4)	C1A'—C12'—C13'—C14'	0.3 (7)
C9A—C10A—C11A—C16	2.0 (7)	C18'—N2'—C14'—C15'	−174.4 (4)
C16—C11A—C12A—C13	1.2 (7)	C17'—N2'—C14'—C15'	1.3 (6)
C10A—C11A—C12A—C13	179.3 (4)	C18'—N2'—C14'—C13'	6.4 (6)
C11A—C12A—C13—C14	−1.4 (7)	C17'—N2'—C14'—C13'	−177.8 (4)
C17—N2—C14—C13	178.7 (4)	C12'—C13'—C14'—N2'	178.9 (4)
C18—N2—C14—C13	−5.1 (6)	C12'—C13'—C14'—C15'	−0.2 (6)
C17—N2—C14—C15	−0.6 (6)	N2'—C14'—C15'—C16'	−179.7 (4)
C18—N2—C14—C15	175.6 (3)	C13'—C14'—C15'—C16'	−0.6 (6)
C12A—C13—C14—N2	−178.4 (4)	C14'—C15'—C16'—C1A'	1.3 (7)
C12A—C13—C14—C15	0.9 (6)	C12'—C1A'—C16'—C15'	−1.2 (7)
N2—C14—C15—C16	178.9 (4)	C0'A—C1A'—C16'—C15'	178.5 (5)
C13—C14—C15—C16	−0.4 (6)	C5'B—C4'B—C9'B—C0'B	175 (2)
C14—C15—C16—C11A	0.2 (6)	C3'B—C4'B—C9'B—C0'B	−5 (4)
C12A—C11A—C16—C15	−0.6 (6)	C4'B—C9'B—C0'B—C1B'	−178 (3)
C10A—C11A—C16—C15	−178.6 (4)	C5B—C4B—C9B—C10B	17 (15)
C2'—N1'—C1'—C7'	−101.2 (5)	C3B—C4B—C9B—C10B	−167 (10)
C6'—N1'—C1'—C7'	76.9 (5)	C4B—C9B—C10B—C11B	−166 (10)
C6'—N1'—C2'—C3'A	2.2 (7)	C9B—C10B—C11B—C12B	−53 (13)
C1'—N1'—C2'—C3'A	−179.7 (4)		

### Hydrogen-bond geometry (Å, º)

**Table:**

*D*—H···*A*	*D*—H	H···*A*	*D*···*A*	*D*—H···*A*
O1—H1*O*···Cl1	0.83 (3)	2.20 (3)	3.024 (3)	174 (4)
O2—H2*O*···Cl1^i^	0.83 (3)	2.27 (3)	3.086 (3)	169 (4)
C2—H2···O2′^i^	0.95	2.40	3.222 (5)	145
C5*A*—H5*A*···Cl2^ii^	0.95	2.78	3.687 (5)	159
C8—H8*A*···N2^ii^	0.99	2.65	3.637 (5)	176
O1′—H1*O*′···Cl2	0.84 (3)	2.25 (3)	3.083 (3)	171 (4)
O2′—H2*O*′···Cl2^iii^	0.83 (3)	2.29 (3)	3.104 (3)	169 (4)
C6′—H6′···O2^iii^	0.95	2.40	3.236 (6)	147

Symmetry codes: (i) *x*+1, *y*, *z*; (ii) −*x*+1, *y*−1/2, −*z*+1; (iii) *x*−1, *y*, *z*.
